# Low-density lipoprotein cholesterol levels are associated with insulin-like growth factor-1 in short-stature children and adolescents: a cross-sectional study

**DOI:** 10.1186/s12944-019-1062-z

**Published:** 2019-05-24

**Authors:** Qianqian Zhao, Yingzhe Jiang, Mei Zhang, Yuntian Chu, Baolan Ji, Hui Pan, Bo Ban

**Affiliations:** 1 Department of Clinical Medicine, Jining Medical University, 16 Hehua Road, Beihu New District, Jining, Shandong 272067 People’s Republic of China; 2 Department of Endocrinology, Affiliated Hospital of Jining Medical University, Jining Medical University, 89 Guhuai Road, Jining, Shandong 272029 People’s Republic of China; 3 School of Health Management and Medicine, Tongji Medical College, Huazhong University of Science and Technology, Wuhan, Hubei 430030 People’s Republic of China; 4 Key Laboratory of Endocrinology of National Health and Family Planning Commission, Department of Endocrinology, Peking Union Medical College Hospital, Chinese Academy of Medical Science and Peking Union Medical College, Beijing, 100730 People’s Republic of China; 5 Chinese Research Center for Behavior Medicine in Growth and Development, 89 Guhuai Road, Jining, 272029 Shandong People’s Republic of China

**Keywords:** Insulin-like growth factor-1, Low-density lipoprotein cholesterol, Coronary heart disease, Short stature

## Abstract

**Background:**

Elevated low-density lipoprotein cholesterol (LDL-C) levels in childhood have recently been found to be the strongest predictive risk factor for coronary artery disease in adulthood. There is an increased level of LDL-C in children and adolescents with short stature. However, the underlying factors associated with increased LDL-C levels in children and adolescents with short stature are unknown. In addition, the insulin-like growth factor 1 (IGF-1) level in the short-stature population is usually below the normal reference range. The aim of this study was to investigate the relationship between IGF-1 standard deviation score (IGF-1 SDS) and LDL-C level in children and adolescents with short stature.

**Methods:**

A cross-sectional study was conducted in a single centre of China, 557 short-stature children and adolescents whose height SDS was lower than − 2 SD after adjustment for age and gender were included. The related clinical and laboratory examinations, including anthropometric parameters, lipid profiles, IGF-1 levels and the levels of other cofactors, were assessed in all participants.

**Results:**

The univariate analysis results showed a significant negative correlation between IGF-1 SDS and LDL-C levels (*P* = 0.006). Furthermore, a nonlinear relationship was observed between IGF-1 SDS and LDL-C by smooth curve fitting after adjusting for possible confounders. A multivariate piecewise linear regression model revealed a significant negative correlation between IGF-1 SDS and LDL-C when the IGF-1 level was greater than − 2 SDS (β − 0.07, 95% CI -0.12, − 0.02; *P* = 0.006). However, we did not observe a significant relationship between IGF-1 SDS and LDL-C when the IGF-1 level was lower than − 2 SDS (β 0.08, 95% CI -0.02, 0.17; *P* = 0.119).

**Conclusion:**

This study demonstrated a nonlinear relationship between IGF-1 and LDL-C independent of other potential confounding factors, suggesting that circulating IGF-1 may contribute to the regulation of LDL-C levels, thus meriting further investigation.

**Electronic supplementary material:**

The online version of this article (10.1186/s12944-019-1062-z) contains supplementary material, which is available to authorized users.

## Introduction

Dyslipidaemia in childhood and adolescence is a strong marker of atherogenic risk and may contribute to the development of coronary heart disease (CHD) in adulthood [1, 2]. Childhood atherosclerosis may not be obvious, but atherosclerosis is a multifactorial disease with its roots in childhood [3, 4]. Endocrinologists have paid considerable attention to screening lipid profiles and have demonstrated the related factors in youths, especially in obese children, who are prone to having unfavourable lipid profiles [5–7]. Some studies have provided evidence that short stature is a risk factor associated with CHD [8, 9] and non-high-density lipoprotein (non-HDL) cholesterol levels even after adjustment for body mass index (BMI) in adults [10], and recent studies have reported that an inverse relationship between height and non-HDL cholesterol is also exists in children [11–13]. Therefore, these studies suggest the need to analyse the lipid profiles and the associated factors, especially in children and adolescents with short stature.

The accumulation of low-density lipoprotein cholesterol (LDL-C), which forms fatty streaks in the intima is the earliest morphological change occurring in the development of atherosclerosis [14, 15]. Previous longitudinal prospective cohort studies have demonstrated that compared to the other lipid indexes, LDL-C evaluated in childhood is the strongest predictor for increased risk of adulthood dyslipidaemia and CHD [16, 17]. The assessment of childhood CHD risk based on LDL-C may be useful for preventing cardiovascular disease in adulthood [16]. Furthermore, a recent study reported that a normal LDL-C levels were associated with subclinical atherosclerosis in a middle-aged population of 4184 adults aged between 40 and 54 years of age [18]. The LDL-C level is typically multifactorial, and it is well-established that BMI and LDL-C have a positive relationship in obese children [19–21] and non-obese children [22–24]. Children and adolescents with short stature have increased LDL-C levels [11], but this relationship has not received much attention. A prospective cohort study with 20 years of follow-up has reported that the childhood LDL-C levels are useful for predicting adult dyslipidaemia and other cardiovascular risks [25]. Furthermore, Nelson CP et al. performed a pathway analysis of height-associated factors and identified a primary association between a genetically determined short stature and an increased risk of CHD, which is partially explained by the association between short stature and an adverse lipid profile [26].

As a growth factor, insulin-like growth factor-1 (IGF-1) also plays an important role in the lipid metabolism [27]. According to De Ita JR et al., IGF-1-deficient mice exhibit dysregulated expression of lipid metabolism-related genes and genes encoding enzymes involved in cholesterol synthesis in the liver [28]. A cross-sectional observation in the large-scale community based Framingham Heart Study demonstrated that lower IGF-1 levels are associated with metabolic syndrome [29]. Additionally, an intervention study have demonstrated that short term treatment with recombinant human growth hormone (rhGH) improved lipid metabolism [30]. Moreover, the IGF-1 level in the short-stature population is usually below the normal reference range [31].

Previous studies have shown a controversial relationship between IGF-1 and LDL-C [32] and there was a lack of a database of children and adolescents with short stature. Here, we screened the lipid profiles of children and adolescents with short stature at a single centre in China and investigated the relationship between IGF-1 and LDL-C levels in this population.

## Methods

### Subjects

The study group consisted of 557 children and adolescents with short stature (409 males and 148 females, from 3 to 18 years of age) who were enrolled from May 2013 to December 2017. A retrospective cross-sectional study was carried out at the Department of Endocrinology, Affiliated Hospital of Jining Medical University, Jining, Shandong, China. The subjects were selected based on the following inclusion criteria: short stature, which is defined as a condition in which the individual’s height is more than two standard deviations (SD) below the population mean for the relevant age and gender [35]; normal weight and height at birth; and the absence of chronic disease. The exclusion criteria included children with chromosomal abnormalities, skeletal dysplasia, genetic metabolic diseases, thyroid dysfunction, or abnormal liver function.

### Anthropometric measurement

Anthropometric measurements included measurements of height, weight, systolic blood pressure (SBP), diastolic blood pressure (DBP), and pubertal stage. Height and weight were assessed according to standard procedures with the participants in light clothing, with no shoes. Body height was measured to the nearest 0.1 cm by using a Best Industrial Stadiometer (Nantong Best Industrial Co, Ltd., Jiangsu; China). A scale with a capacity of 120 kg (Wuxi Weigher Factory Co, Ltd., Jiangsu; China) was used to measure body weight to the nearest 0.1 kg. Height and weight were expressed as the standard deviation scores (SDS) based on normative values for Chinese children [36]. BMI was calculated as weight divided by height in metres squared, and the BMI SDS was calculated according to 2009 growth charts for Chinese children and adolescents [37]. Pubertal stage was evaluated by physical examination according to the Tanner stages [38]. The criteria for prepuberty were as follows: for boys, testicular volume less than 4 mL with no pubic hair; for girls, no breast development or pubic hair.

### Laboratory measurements

Fasting blood samples were obtained from all participants to measure the serum IGF-1 level and other laboratory parameters.

Serum IGF-1 concentrations were estimated based on a chemiluminescence assay (DPC IMMULITE 1000 analyser, SIEMENS, Germany) with an intra-assay and inter-assay coefficient of variation of 3.0 and 6.2%, respectively; the IGF-1 SDS, adjusted for age and sex, was calculated according to IGF-1 levels of healthy Japanese children and adolescents of the same age and sex [39]. Total cholesterol (TC), high-density lipoprotein cholesterol (HDL-C), LDL-C, very low density lipoprotein cholesterol (VLDL-C), triglyceride (TG), alanine aminotransferase (ALT), fasting plasma glucose (FPG) and uric acid (UA) levels were determined using an auto biochemical analyser (Cobas c702, Roche; Shanghai, China). LDL-C dyslipidaemia was defined according to an expert consensus regarding Chinese child and adolescent dyslipidaemia [40].

### Statistical analysis

The continuous variables are expressed as the means ± standard deviations, and the categorical data are expressed as a number (percentage). A univariate analysis model was used to determine the significance of the association between LDL-C and IGF-1 SDS as well as the other independent variables. We then investigated the relationship between LDL-C and IGF-1 SDS using smooth curve fitting after adjusting for potential confounders. Finally, we further applied a multivariate piecewise linear regression model to examine the threshold association of LDL-C and IGF-1 SDS. Statistical significance was indicated by as a two-sided *P* value < 0.05. All analyses were performed using EmpowerStats (http://www.empowerstats.com, X&Y Solutions, Inc., Boston, MA) and R 3.4.3 (http://www.r-project.org).

## Results

### Clinical and laboratory characteristics of the subjects

The clinical characteristics of all participants were described in Table 1. A total of 557 children and adolescents with short stature and a mean age of 10.1 ± 3.5 years old were included in the study. The mean height SDS of the participants was − 2.72 ± 0.62. Of the subjects, 409 (73.43%) children were male. The majority of the children, 421 (75.58%) were prepubescent. The mean IGF-1 SDS and LDL-C level were − 1.07 ± 1.31 and 2.06 ± 0.54 mmol/L, respectively. The rate of LDL-C dyslipidaemia in the children and adolescents with short stature was 1.97%, and the rate of occurrence of critical LDL-C dyslipidaemia was 4.13%.

**Table 1:** Clinical and laboratory characteristics of the subjects

Characteristics	All
N	557
Age (years)	10.1 ± 3.5
Male/Female (N)	409/148
Prepuberty/Puberty (N)	421/136
Height (cm)	124.81 ± 17.67
Height SDS	−2.72 ± 0.62
Weight (kg)	27.18 ± 10.36
BMI (kg/m^2^)	16.80 ± 2.78
BMI SDS	−0.28 ± 1.17
IGF-1 (ug/L)	179.80 ± 121.64
IGF-1 SDS	−1.07 ± 1.31
SBP (mmHg)	104.12 ± 11.63
DBP (mmHg)	62.81 ± 8.86
FPG (mmol/L)	4.80 ± 0.68
ALT (U/L)	16.32 ± 9.05
UA (umol/L)	265.76 ± 71.18
TG (mmol/L)	0.71 ± 0.33
TC (mmol/L)	3.84 ± 0.69
HDL-C (mmol/L)	1.36 ± 0.30
VLDL-C (mmol/L)	0.43 ± 0.26
LDL-C (mmol/L)	2.06 ± 0.54
Critical value of LDL-C, N (%)	23 (4.13%)
High value of LDL-C, N (%)	11 (1.97%)

**Abbreviations:** Height SDS: height standard deviation scores; BMISDS: body mass index standard deviation scores; IGF-1 SDS: insulin like growth factor-1 standard deviation scores; SBP: systolic blood pressure, DBP: diastolic blood pressure; FPG: fasting plasma glucose; ALT: alanine aminotransferase; UA: uric acid; TG: triglyceride; TC: total cholesterol; HDL-C: high density lipoprotein-cholesterol; LDL-C: low density lipoprotein cholesterol; VLDL-C: very low density lipoprotein-cholesterol. According to children and adolescents dyslipidemia consensus. Critical value of LDL-C was 2.85–3.34 mmol/L; High value was defined as of LDL-C level ≥ 3.37 mmol/L. Continuous variables are expressed as means ± standard deviations and categorical data using number (percentage)

### Factors correlated with LDL-C in the subjects

Univariate linear regression analysis was performed to determine the relationships between clinical parameters and LDL-C. As shown in Table 2, for the unadjusted model, we observed a significant negative correlation between IGF-1 SDS and LDL-C (*P* = 0.006). Other variables that remained significantly associated with LDL-C were age, weight, BMI SDS, TG, TC, HDL-C, VLDL-C and pubertal stage (*P* < 0.05). No significant association was observed between LDL-C and sex, height SDS, SBP, DBP, FPG, ALT or UA (*P* > 0.05).

**Table 2:** Factors correlated to LDL-C by a univariate analysis

	β	(95% CI)	*P* value
Age (years)	−0.03	(− 0.04, − 0.02)	<0.001
Height SDS	0.04	(− 0.03, 0.11)	0.265
Weight (kg)	0.00	(−0.01, − 0.00)	0.034
BMI SDS	0.07	(0.04, 0.11)	<0.001
IGF1 SDS	−0.05	(− 0.08, − 0.01)	0.006
SBP (mmHg)	− 0.00	(− 0.01, 0.00)	0.507
DBP (mmHg)	−0.00	(− 0.01, 0.00)	0.712
FPG (mmol/L)	−0.06	(− 0.13, 0.01)	0.115
ALT (U/L)	0.00	(−0.00, 0.01)	0.119
UA (umol/L)	0.00	(−0.00, 0.00)	0.456
TG (mmol/L)	0.18	(0.05, 0.32)	0.008
TC (mmol/L)	0.64	(0.61, 0.68)	<0.001
HDL-C (mmol/L)	0.15	(0.00, 0.30)	0.046
VLDL-C (mmol/L)	0.20	(0.02, 0.37)	0.027
Sex			
Male	0		
Female	0.06	(−0.04, 0.16)	0.222
Pubertal stage			
In prepuberty	0		
In puberty	−0.14	(−0.25, − 0.04)	0.007

Abbreviations: Height SDS: height standard deviation scores; BMISDS: body mass index standard deviation scores; IGF-1 SDS: insulin like growth factor-1 standard deviation scores; SBP: systolic blood pressure, DBP: diastolic blood pressure; FPG: fasting plasma glucose; ALT: alanine aminotransferase; UA: uric acid; TG: triglyceride; TC: total cholesterol; HDL-C: high density lipoprotein-cholesterol; LDL-C: low density lipoprotein cholesterol; VLDL-C: very low density lipoprotein-cholesterol. *P* < 0.05 is considered to be statistically significant

### Independent correlation between IGF-1 SDS and LDL-C by multivariate piecewise linear regression

**Fig. 1:**
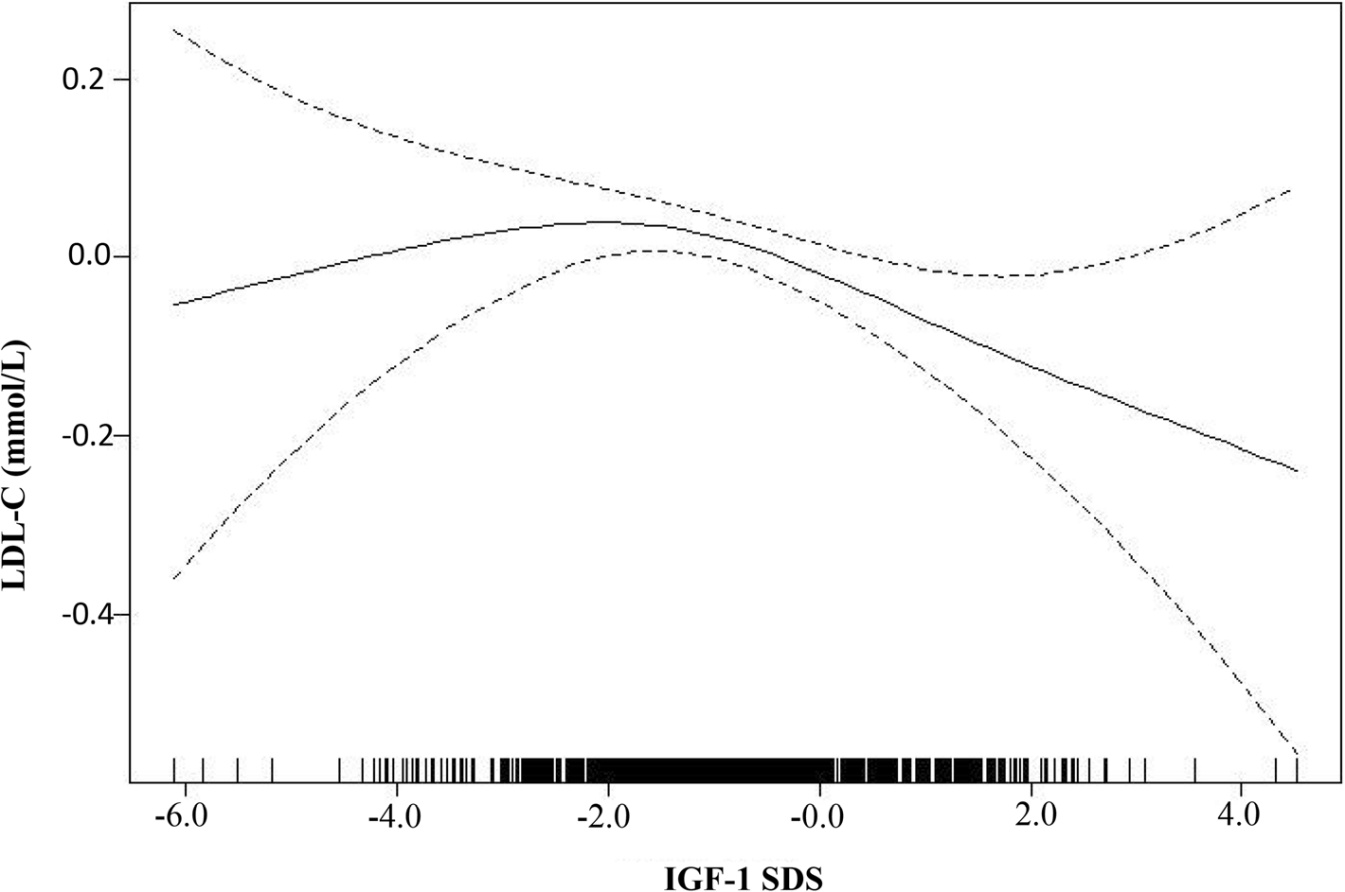
The relationship between IGF-1 SDS and LDL-C by smooth curve fitting. Adjustment variables: weight, BMI SDS, TG, HDL-C, VLDL-C, pubertal stage

As shown in Fig. 1, smooth curve fitting was performed after adjusting for possible confounding factors, including weight, BMI SDS, TG, HDL-C, VLDL-C and pubertal stage. The participants’ LDL-C levels exhibited an nonlinear relationships with IGF-1 SDS and the resulting curve exhibited a two-stage change and a breakpoint. When the IGF-1 SDS level was greater than the breakpoint, there was an inverse relationship between IGF-1 SDS and LDL-C; however, if the value was less than the breakpoint, there was a positive relationship between IGF-1 SDS and LDL-C. As shown in Table 3, we further analysed the threshold effect based on curve fitting, and the data indicated that the inflection point of the IGF-1 SDS was − 2. Specifically, LDL-C levels decreased as the IGF-1 SDS increased when the IGF-1 SDS was greater than − 2 (β − 0.07, 95% CI -0.12, − 0.02; *P* = 0.006). However, the LDL-C levels displayed an increasing trend as the IGF-1 SDS increased when the IGF-1 SDS was less than − 2, but the difference was not statistically significant (β 0.08, 95% CI -0.02, 0.17; *P* = 0.119). In addition, as shown in Additional file 1: Table S1, after stratification by pubertal stage, a nonlinear relationship between IGF-1 SDS and LDL-C remained.

**Table 3:** The independent correlation between IGF-1 SDS on LDL-C by multivariate piecewise linear regression

Inflection point of IGF-1 SDS	β (95% CI)	*P* value
< −2.0	0.08 (− 0.02, 0.17)	0.119
> −2.0	− 0.07 (− 0.12, − 0.02)	0.006

**Abbreviations:** IGF-1 SDS: insulin likes growth factor-1 standard deviation scores; LDL-C: low density lipoprotein-cholesterol. Adjusted for weight, BMI SDS, TG, HDL-C, VLDL-C, pubertal stage. *P* < 0.05 is considered to be statistically significant

## Discussion

In this study, we observed that there was a nonlinear relationship between IGF-1 SDS and LDL-C in children and adolescents with short stature, and the IGF-1 SDS turning point was − 2. The negative relationship between IGF-1 SDS and LDL-C was significant only when the IGF-1 SDS reached the inflection point.

In the children and adolescents with short stature, we observed that the IGF-1 SDS was a protective factor against elevated LDL-C levels. This result was consistent with the findings of previous clinical studies, a study conducted in women with primary hypothyroidism showed that IGF-1 was a determinant of the concentration of LDL-C and that there was a negative relationship between IGF-1 and LDL-C in individuals with hypothyroidism [32]. In addition, there is a similar relationship between IGF-1 and LDL-C in mildly hypercholesterolemic women [33]. However, this relationship was not significant in a study carried out in healthy subjects [34], and the differences in the clinical characteristics and recruitment criteria among these studies may explain this divergent result. Furthermore, we revealed a nonlinear relationship between IGF-1 SDS and LDL-C, suggesting that a certain IGF-1 level should be maintained to modulate the serum LDL-C concentration. The potential mechanisms of this phenomenon can be explained as follows: IGF-1 can downregulate 12/15-lipoxygenase, thereby suppressing lipid oxidation [41], and decreased IGF-1 may impair the expression of genes involved in lipid catabolism and dysregulate the expression of related proteins encoding low-density lipoprotein receptors [28]. Furthermore, it was found that the level of LDL-C is decreased in children with growth hormone deficiency (GHD) as a result of rhGH replacement therapy. These findings support the notion that IGF-1 plays a relevant role in modulating the serum LDL-C concentration.

Obese children are prone to dyslipidaemia, and our study also showed that the BMI SDS had a positive relationship with LDL-C. This finding was consistent with the results of a previous study reporting a positive relationship between LDL-C and BMI in a large population-based sample of Japanese school children [42]. Furthermore, serum lipid levels and BMI in childhood correlate strongly with these values in middle age [43]. The correlation between short stature and elevated plasma LDL-C levels has also been observed in school children in fifth grade [11], however, we did not find an association between LDL-C and height, which may be because the study subjects with a height SDS of no more than − 2 were limited.

In addition, this study also revealed a negative relationship between age, pubertal stage and LDL-C. Namely, the data show small decreases in LDL-C as age increases, which is consistent with a decreases in LDL-C levels occurring along with advancing sexual development. This finding was consistent with the recent study by Eissa that showed that non-HDL levels decrease with progressing Tanner stage [44].

The relationship between IGF-1 SDS and LDL-C has been considered in only a few papers. In our report, we confirm that this relationship can be observed in children and adolescents with short stature. The level of IGF-1 level is related not only to growth but also to lipid metabolism.

The present study has several limitations. The first limitation is that the cross-sectional design of this study does not allow us to determine causality. Second, the negative relationship between LDL-C and IGF-1 SDS needs further validation in a prospective study. Third, further investigation is necessary to follow up on age changes to determine whether the relationship between IGF-1 and LDL-C would remain as individuals age. Additionally, additional research is needed to fully understand the mechanism underlying the association between IGF-1 and LDL-C.

## Conclusion

In conclusion, this study described a nonlinear relationship between IGF-1 and LDL-C levels in children and adolescents with short stature after adjusting for potential confounders. This finding suggests that in children and adolescents with short stature, the level of IGF-1 is not related only to its growth effect, but also to its lipid metabolism effects.

## Additional file

Additional file 1: **Table S1.** The independent correlation between IGF-1 SDS and LDL-C levels identified using multivariate piecewise linear regression analysis in groups stratified according to before and after puberty. (DOC 28 kb)

## Abbreviations

ALT: alanine aminotransferase
BMISDS: body mass index standard deviation scores
CHD: coronary heart disease
DBP: diastolic blood pressure
FPG: fasting plasma glucose
HDL-C: high density lipoprotein-cholesterol
Height SDS: height standard deviation scores
IGF-1 SDS: insulin like growth factor-1 standard deviation scores
LDL-C: low density lipoprotein cholesterol
non-HDL: non-high density lipoprotein.
SBP: systolic blood pressure
TC: total cholesterol
TG: triglyceride
UA: uric acid
VLDL-C: very low density lipoprotein-cholesterol
