# Supplementary material for: Low-density lipoprotein cholesterol levels are associated with insulin-like growth factor-1 in short-stature children and adolescents: a cross-sectional study
Source: Lipids Health Dis. 2019 May 24;18:120. doi: 10.1186/s12944-019-1062-z (PMC6533685; doi:10.1186/s12944-019-1062-z)
Supplement: Supplementary file 1 — Table S1. The independent correlation between IGF-1 SDS and LDL-C levels identified using multivariate piecewise linear regression analysis in groups stratified according to before and after puberty. (DOC 28 kb) [file 12944_2019_1062_MOESM1_ESM.doc]

**Additional file 1**: **Table S1** The independent correlation between IGF-1 SDS and LDL-C levels identified using multivariate piecewise linear regression analysis in groups stratified according to before and after puberty.

| Inflection point of IGF-1 SDS | β 95% CI | P value |
| --- | --- | --- |
| In prepuberty |  |  |
| < -2.0 | 0.07 (-0.03, 0.17) | 0.152 |
| > -2.0 | -0.07 (-0.12, -0.01) | 0.023 |
| In puberty |  |  |
| < -2.0 | -0.08 (-0.38, 0.23) | 0.621 |
| > -2.0 | -0.11 (-0.20, -0.02) | 0.019 |

**Abbreviations:** IGF-1 SDS: insulin-like growth factor-1 standard deviation score; LDL-C: low-density lipoprotein-cholesterol. Adjusted for weight, BMI SDS, TG, HDL-C, VLDL-C. P < 0.05 is considered statistically significant.
